# “Who's breaking the law … not us, them!”: Inside immigration detention in Portugal

**DOI:** 10.1002/ajcp.12784

**Published:** 2025-02-13

**Authors:** Francesca Esposito, Federica de Lellis, Federica De Cordova, Erica Briozzo

**Affiliations:** ^1^ Department of Psychology “Renzo Canestrari” University of Bologna Cesena Italy; ^2^ Centre for Social Justice Research (CSJR) University of Westminster London UK; ^3^ Department of Psychology Università degli Studi di Milano Bicocca Milan Italy; ^4^ Department of Human Sciences Università di Verona Verona Italy; ^5^ Department of Applied Psychology Research Center Capability and Inclusion (APPsyCI‐FCT/UIDB/05299/2020) Ispa‐Instituto Universitário de Lisboa Lisbon Portugal

**Keywords:** border violence, (cis and trans) women, immigration detention, intersectionality, justice‐centered community psychology ecological framework, Portugal, survival and resistance

## Abstract

In this paper, we examine immigration detention in Portugal, a system whose daily operations and inherent violence are overlooked in both public and academic discourses. Even within community psychology, discussions on immigration detention have largely remained on the fringes of scholarly debates. Guided by a justice‐centered ecological lens, we map the contours of daily life in detention by centering the intersectional struggles of detained cisgender and transgender women. These struggles illuminate the politics of power and resistance at play in these sites, contributing to interrelated ecologies of knowledge and advancing a critical understanding of the systems of power and oppression articulated around borders, citizenship, and the “making of migration.” Our findings reveal Portuguese detention centers as *uncaring* environments where women feel constantly threatened, unsafe and disregarded, with their well‐being severely compromised. Ignorance reigns in these sites, wielded as a form of power. Yet, despite this, detained women create counterspaces and cultivate ecologies of knowledge and resistance from the ground up. We conclude by reflecting on how community‐engaged scholars and activists can contribute to transformative and liberatory efforts against carceral border systems, working toward futures of freedom, dignity, and justice for all.

## Highlights

- Psychological clinical analyses of detention risks individualizing systemic oppression.
- Detention centers are *uncaring* spaces where women feel unsafe, threatened, and their wellbeing is compromised.
- A politics of ignorance blocks information flow and hinders effective intersectional solidarities.
- Through action‐research, we can bear witness and amplify demands for justice, freedom, and detention abolition.

The 21st century has witnessed a renewed anxiety about borders and their control, accompanied by a growing state reliance on violent strategies to filter and manage people attempting to cross them. Such measures have often taken various forms of containment, with immigration detention being one of the most extreme. Immigration detention is the practice of depriving individuals of their liberty based solely on their migration status (Flynn, 2012). This migration‐related incarceration is typically enforced “without the normal due process safeguards commonly demanded in liberal democracies” (Wilsher, 2011, p. ix). Targeting individuals classified as noncitizens, detention plays a crucial role in determining who is considered part of the national community and who is not, thereby “enforcing and creating degrees of belonging and alien‐ness in the project of nation‐building” (Bashford & Strange, 2002, p. 10). This illustrates how the notion of citizenship, along with the exclusionary hierarchies of membership it relies on, underpins the day‐to‐day operation of carceral border regimes.

Despite growing public and scholarly interest in border control and detention policies and their effects, little is known about daily life inside immigration detention centers, as these highly politicized and contested sites remain difficult for researchers to access (Bosworth, 2014; Kirmayer et al., 2004). Empirically informed analyses are particularly scant, and data is almost nonexistent in some countries, such as Portugal. In psychology, the literature on immigration detention and deportation is limited, and existing studies have often adopted a clinical approach that focuses on detained individuals' suffering (Von Werthern et al., 2018). While these studies highlight the immense human costs of detention, their reliance on medicalized language risks reinforcing an individualistic focus that overlooks the complex struggles of detained people and the broader social and political contexts contouring them (Esposito et al., 2019). In doing so, they also undermine the political agency of those detained by confining them to a passive “sick role” (McGregor, 2011). Even within community psychology, a discipline that has historically critiqued the individual‐level emphasis characterizing psychological research, discussions on immigration detention have largely remained on the fringes of scholarly debates (for exceptions, see Chicco et al., 2016; Esposito et al., 2019; Langhout et al., 2018). In contrast, existing scholarship has primarily focused on the needs and experiences of migrants and refugees regarding integration and acculturation processes (e.g., Jurcik et al., 2019; Zou & Deng, 2021).

In this paper, we address this knowledge gap by examining the Portuguese detention regime, whose daily operations and related violence are largely absent from both public and scholarly discussions. Hence, we add to various, although interrelated, ecologies of knowledge. First, the paper contributes to activist and scholarly knowledge of immigration detention and, particularly, to empirical analyses of daily life within these custodial sites. More specifically, it adds to the limited research centering the intersectional experiences of (cis and trans) women within these sites (for exceptions, see Bachmann, 2016; Bosworth et al., 2018; Bosworth & Kellezi, 2014; Canning, 2017; Esposito et al., 2020; Human Right Watch, 2016; Lousley & Cope, 2017).^1^ Second, we expand the application of a justice‐centered community psychology ecological framework previously developed by some of us and used to examine other detention settings (Esposito et al., 2019). This analytical framework moves beyond the reductionism of individual‐centered psychological research by emphasizing the intersectional experiences of detained people as a crucial asset in building systems knowledge from the ground up. Doing so, it highlights the complex relationships between power, violence, and resistance within these confinement sites. Finally, and importantly, our analysis forms part of a broader effort to advance a critical understanding of systems of power and oppression articulated around borders, citizenship, and “the making of migration” (Tazzioli, 2020) within the discipline of community psychology (Esposito et al., 2024). Such efforts acknowledge people's lived experiences of the border not as mere “objects of investigation” but as a vital lens through which to unpack the violent operation of carceral border regimes.

Inspired by these goals, the questions we aim to address in this paper are as follows: What are the oppressive qualities of immigration detention, and what complex struggles do people face in these settings? How do the intersectional experiences of (cis and trans) women illuminate the intricate workings of these systems? What multifaceted strategies do women employ to navigate, survive, and contest the uncertain and volatile environment of detention? More importantly, what do their accounts of daily life in these custodial sites reveal about the politics of power and resistance at play? Ultimately, we hope the critical insights generated through this analysis will spark essential discussions on how community‐engaged scholars, activists, and practitioners can contribute to transformative and liberatory efforts against carceral border systems, working toward futures of freedom, dignity, and justice for all.

## IMMIGRATION DETENTION IN PORTUGAL

Organic knowledge of immigration detention in Portugal is rather scarce, supported by the lack of official data on how many people enter this system annually. In this scenario, the country's public representation of immigration policies is based on two polarities. On the one hand, Portugal enjoys a reputation as a welcoming, progressive country (Solano & Huddleston, 2020). On the other hand, critical researchers, journalists, and activists have provided evidence of the widespread use of border violence, particularly regarding detention and deportation enforcement (e.g., Gorjão Henriques, 2018, 2019). The violence of the Portuguese border regime and the harshness of detention conditions were starkly revealed in March 2020 with the death of Ukrainian citizen Ihor Homeniuk at the hands of three border guards inside Lisbon Airport's “waiting zone” (Demony, 2021). However, the way media framed the debate left no room for questioning the inherently violent nature of nation‐state borders or their role in the (re)production of geopolitical power hierarchies (Garraio et al., 2022). Critical scholars have also highlighted the colonial underpinnings of the racialized (classed and gendered) state‐sanctioned violence at Portuguese borders, along with the “politics of organized forgetting” that sustains it (Esposito & Caja, 2025).^2^

Immigration detention in Portugal dates back to 1994—when Law 34/94 was enacted, establishing the creation of ad hoc facilities for detaining individuals under immigration powers. This legislation led to the establishment of five centers euphemistically named *Espaços Equiparados a Centros de Instalação Temporária* (EECITs, Spaces Equivalent to Temporary Installation Centers) at the country's main airports: Lisbon, Porto, Faro, Funchal, and Ponta Delgada.^3^ These centers, like similar ones inside international airports, are internationally known as “waiting zones,” and their primary goal is to enforce refusal of entry at external borders. Yet in practice, EECITs in Portugal have been used for various purposes, particularly for detaining individuals for up to 60 days (the statutory maximum duration, extendable to 90 days in special circumstances and with a judicial order, Art. 160, Immigration Act 1965). As mentioned earlier, there is no official data on the number of people who enter Portuguese airport “waiting zones.” However, some of us determined that, in 2020 alone, 1353 people entered the Lisbon EECIT, despite being closed from March to August (Esposito & Caja, 2025). This suggests the number of people detained annually in this site is likely much higher under normal circumstances.

In 2006, a *Centro de Instalação Temporária* (CIT, Temporary Installation Center; hereinafter, *Unidade Habitacional de Santo António* [UHSA]) was opened in Porto—becoming the sole immigration detention center in the country, according to EU‐wide standards. Our research took place at this center, which can hold up to 30 adults and six children. From 2017 to 2022, 981 adults and 36 minors were detained at this site, with annual figures ranging from 130 to 203 people overall (Esposito & Caja, 2025). Most of those detained were identified as men, with only 157 women recorded during this period.^4^

Managed by the state through its immigration enforcement police,^5^ these centers rely on cooperation protocols with nonstate actors for their day‐to‐day operations. In the case of the UHSA, external partners include the *Serviço Jesuíta aos Refugiados* (SJR, Jesuit Refugee Service), which provides psychosocial and legal support; *Médicos do Mundo* (MdM, Doctors of the World), which offers medical assistance; and the International Organization for Migration (IOM Portugal, n.d), responsible for training detention staff, monitoring human rights, and “inform[ing] detained migrants on their rights, duties, and guarantees and how to migrate regularly.” For their activities, these external partners are funded either partially or fully by the Ministry of the Interior and its immigration enforcement agencies. Additionally, in each center, 24‐h security personnel from a private security company (currently *Securitas Portugal*) are employed under a contract with the immigration enforcement police.

Like in other jurisdictions, different people can be confined in these sites, including illegalized migrants,^6^ people seeking asylum, and individuals with experiences with the criminal punishment system. In Portugal, those labeled as “vulnerable” can also be detained—such as victims of trafficking and torture, the elderly, people with complex (physical and mental) health issues and disabilities, pregnant women, and children.

## METHODOLOGY

### Situating ourselves and our research engagement

Driven by a commitment to transformative, social justice‐informed research, we, the authors of this paper, engage in a critical analysis of carceral border regimes in all their violent forms, including immigration detention. Our aim is to challenge these systems of power and oppression and expose the normalization of border violence and injustice (Dutta et al., 2016; Farmer, 2013). Therefore, our research and writing praxis is deeply informed by migrant solidarity and border abolitionist struggles (Riva et al., 2024). Thus, we oppose the ontologization of the “migrant” (Scheel & Tazzioli, 2022), acknowledging the entanglement of racialization and migrantization and their social productive nature (Dutta et al., 2024; Esposito et al., 2024).^7^

Alongside other critical community psychology scholar‐activists (e.g., Dutta et al., 2024; Lykes et al., 2011), we engage in a praxis of “psychosocial accompaniment” (Watkins, 2015), bearing witness to the struggles of those at the sharp end of violent border regimes. Informed by liberation psychology principles, psychosocial accompaniment represents an ethical and political commitment to walk alongside individuals and communities most exposed to abuse, violence, torture, and premature death. This approach highlights the need to learn from their experiences, bear witness, practice solidarity, speak truth to power, and stand with them for justice (Edge et al., 2003).

Psychosocial accompaniment in psychological research goes beyond focusing solely on the individual. It requires a commitment to uncovering the sociopolitical roots of people's complex suffering, decolonizing one's mindset and practices, and creating dialogic knowledge that supports struggles for justice and liberation. Achieving this, demands pairing structural analyses with insights into the affective significance of lived experiences of oppression (Farmer, 2013). In our work, this means that a critical understanding of the sociopolitical and historical contexts of immigration detention and the ecologies of power in which this system is embedded must be combined with an active listening to the lived experiences and perspectives of those confined in these oppressive institutions. This onto‐epistemic and relational standpoint (Dutta et al., 2024) subverts traditional ways knowledge creation is conceived in Western mainstream academia, interrogating hegemonic research norms, practices, and the power structures they are embedded in. Relationships formed in this context extend beyond traditional “researcher‐participant” roles, instead being cultivated through a productive interplay of activism, research, collaboration, and solidarity.

This standpoint also guided the critical research engagement (2015–2021) underpinning this article.^8^ The fieldwork was conducted by Francesca, the only one granted permission to enter the UHSA. Practicing psychological accompaniment in such an oppressive context as a detention center is no easy endeavor and requires a continual interrogation of the power and privilege held by activist researchers and accompaniers. Firstly, we need to acknowledge that all of us involved in writing this paper are cisgender, White European women, each affiliated to varying degrees with academic institutions.^9^ Some of us identify as part of the LGBTQIA+ community, and some have personally experienced the precariousness of migration and the scrutiny associated with immigration control regimes. However, compared to the women we met in detention, we all enjoy significant privileges and freedoms, including the freedom of movement. In the context of this research, we sought to reposition our privilege by sharing all available information with those we encountered in detention (e.g., regarding rights, laws, procedures, and sources of support both inside and outside detention) and offering emotional proximity and solidarity in their struggles. Rather than turning away from detained individuals—as society often does—ignoring what creates discomfort, practicing accompaniment, as Watkins (2015) suggests, means choosing to engage with these oppressive spaces to break state‐imposed isolation and foster connections with the outside world. This was also our goal, although in a limited capacity. To this end, between 2015 and 2018, Francesca spent significant time at the UHSA, building lasting relationships with detained people and engaging in meaningful discussions about the research's goals and priorities, as well as about existing laws, services, and resources to affirm their rights. Some of these solidarity relationships continue to this day.

### Entering detention

#### Documenting violence from the bottom up

Field observations and in‐depth conversations were used to collect data throughout the research process. Observations, recorded through field notes, focused on significant processes, events, and activities in the detention setting, interactions, and conversations among various actors, meaning‐making processes, topics discussed, physical and environmental characteristics, sensory information, and the researcher's self‐reflections. This was complemented by in‐depth conversations with detained people, aimed at sharing and reflecting on their experiences of life in detention, the affective meanings attached to it, and their critical understanding of the system's workings. Topics covered in these conversations varied but often included daily routines, living conditions, treatment, relationships with detention staff (immigration officers, security personnel, volunteers, nongovernmental organization staff), relationships among detained people, and access to justice and rights. These accounts provided unique insights into the Portuguese detention regime, shedding light on the dynamics of power, violence, survival, and resistance within the detention center where the research took place.

In this paper, we focus on the accounts of the detained (cis and trans) women we encountered, as their intersectional experiences and struggles are often overlooked (Bosworth & Kellezi, 2014). Given the limited number of women entering the Portuguese detention system, no specific selection criteria were applied. All those who identified as women were over 18 years old, and were willing to participate in the research were invited to share their views and experiences. Francesca spoke with 25 women, 23 cis, and two trans women (see Appendix 1). Many of these women had recently arrived in Portugal, having been stopped at the border and subsequently taken into detention. Others had lived in the country for varying lengths, some up to 17 years. Regardless of their backgrounds, detention often resulted in the forced separation of women from their loved ones. Highlighting the colonial continuities in contemporary carceral border regimes, the most common countries of origin among the women we encountered were Brazil and Angola, both former Portuguese colonies. Notably, most of the women we met had initially been detained in the Lisbon “waiting zone” (see “Detained on Arrival” in Appendix 1) before being transferred to the UHSA. In all these cases, their detention at Lisbon Airport exceeded 48 h.

In‐depth conversations were conducted primarily in Portuguese and English, with some in Italian and Spanish, all of which are languages Francesca speaks fluently. In three instances, where the women spoke French or Krio, conversations were facilitated by other detained women or, in one case, by a legal expert supporting the woman's case. The length of the conversations varied, ranging from 35 min to almost 3 h. All women consented to participate in the research and for the conversations to be recorded. These were then transcribed verbatim, anonymized, cleaned of sensitive data, and analyzed using NVivo software.

#### Generating knowledge from protagonists' narratives

We chose to conduct our analysis using a reflexive thematic approach, inspired by Braun and Clarke (2019). This approach was deemed appropriate for capturing the complexities of life in detention and centering the subjectivity of the women, including our own as women researchers. Our engagement with our protagonists' rich accounts combined both top‐down (*theory‐driven*) and bottom‐up (*data‐driven*) approaches. On one hand, the analysis and interpretation were guided by a justice‐centered ecological framework, informed by critical insights from community psychologists (Esposito et al., 2019). We adopted Kelly's (2006) four principles—Interdependence, Cycling of Resources, Adaptation, and Succession—and incorporated Prilleltensky's (2012) principle of Justice. As outlined in Appendix 2, these principles were adapted to understand the ecology of life in immigration detention centers and the complex experiences of those within them. On the other hand, we grounded the broad principles in our framework—serving as overarching themes or “central organizing concepts” (Clarke & Braun, 2017)—in the specific meanings and insights emerging from our protagonists' narratives, thus striving to build systems knowledge from the bottom up. This approach allowed us to shed light on the intricate dynamics of power, violence, and resistance within the context of immigration detention.

At a practical level, we coded conversations and developed themes and subthemes through a collaborative process of data immersion and discussion over several months. This approach drew inspiration from Braun and Clarke's (2006) thematic analysis phases, emphasizing a reflexive and thoughtful engagement with analysis as a fluid, recursive, and collaborative process (Braun & Clarke, 2019). Initially, we read each conversation transcript multiple times, noting key ideas and emerging insights. Summaries were also created to capture the primary aspects of each narrative (Phase 1). Using NVivo software, two team members, Erica and Federica, then conducted preliminary coding guided by the justice‐centered community psychology ecological framework described above. They began by coding the transcripts based on our framework's five principles: Interdependence, Cycling of Resources, Adaptation, Succession, and Justice. Monthly team meetings allowed us to collectively review and agree on these codes, fostering a shared understanding of how the principles applied within the specific context of our research (Phase 2). At this point, we shifted to a data‐driven approach. For each principle, codes were generated through a bottom‐up process, aiming to remain true to the women's perspectives and often incorporating their own words to define these codes. Collective discussions helped us develop a richer, more nuanced reading of the data (Phase 3). Once we had a comprehensive list of codes, Erica and Federica grouped them into potential themes and subthemes (Phase 4). We then created a preliminary thematic map, critically reviewing themes and subthemes with a particular focus on the principles of Justice and Adaptation, which emerged as particularly relevant (Phase 5). This led to a series of collective discussions in which we refined the codes, themes, and subthemes, cultivating a shared understanding of each theme's specific meaning. This recursive and reflective process ultimately enabled us to create a thematic map that accurately reflected the meanings emerging from our analysis (Phase 6). This process was enriched by Francesca's participant observations, which provided valuable context for the insights generated from women's rich accounts. Finally, we selected the excerpts that could compellingly illustrate our protagonists' experiences and perspectives. These excerpts were woven into our analytical narrative, constructing an overall story that conveys a nuanced understanding of the intricate workings of detention and the complex intersectional struggles (cis and trans) women face in these settings (Phase 7).

Below, we present the main findings generated through our analysis, focusing on the principles we identified as most significant: Justice, addressing the violence, abuse, and injustices women experience in detention; and Adaptation, reflecting women's agency, survival, and creative resistance within this oppressive sites.

## RESULTS

### Detention centers as uncaring environments: Governing by ignorance

Immigration detention centers can be described as “total institutions,” in Goffman's (1961) sense. Accordingly, the women we met frequently mentioned hyperregularization and control as defining aspects of life in such a custodial environment. This control pervaded all daily activities, determining when they could get up, access their mobile phones, receive visits, engage in recreational activities, and even eat. Control over food was frequently cited as a major area of concern. Inside the UHSA, meals were scheduled three times a day (breakfast, lunch, and dinner), and outside of these institutionally defined hours, women could not eat, even in special circumstances. This was the case with Delfina, who was pregnant at the time she was detained:

If you didn't have time to come and get food on time, you don't eat… When I arrived, I couldn't get up early because of the cold. So, when I went to ask for breakfast, they said, they said to me I had to wait until 12 p.m. Imagine a pregnant woman staying from morning until 12 p.m. without eating.

[translation by the authors (TbA)]

Overall, women felt a sense of imprisonment because their liberty was taken away and they lost all control over their daily lives. Institutional rules also constrained access to personal items; what women could take with them into the rooms/cells was severely limited, and all other items had to remain in the detention center deposit, being accessible only at specific times and through the mediation of center staff. This situation generated a sense of distress, as described by Makeda: “It's more or less like prison… They take you away your freedom, you do everything according to times, it's tough!.”

Control over one's life is a key determinant of wellness (Marmot, 2004), yet autonomy and self‐determination are systematically denied in immigration detention. At the UHSA, for instance, there were no dedicated spaces for social or religious activities. Women were mixed with men during the day and constantly monitored by both male and female officers, compromising their safety and privacy. As one woman, Emilie, recounted: “Once, three women were showering at the same time. The [male] security officer entered, thinking they were doing something else. He thought they were three lesbians … and then it was that they realized they were being watched” [TbA]. The lack of activities and stimulation intensified the sense of constraint generated by this restrictive environment. Detained people were offered no educational or job opportunities, and recreational activities were limited to occasional, volunteer‐led initiatives (only in the UHSA). As a result, life in detention was perceived as stagnant and stuck. As Priscilla noted, “[Life inside the UHSA is] monotonous. Every day feels the same day.” [TbA]

Alongside control and discipline, abandonment and neglect also emerged as modes of governing people in these sites (see Esposito et al., 2019; Lindberg, 2022). This was especially stark in women's accounts of life in Lisbon's “waiting zone.” Living conditions there, where women often spent considerable time before being transferred to the UHSA, were described as extremely poor. People, often with young children, slept on floor mattresses in overcrowded, unhygienic conditions. No activities were available, and there was no play area for children. As described by Theresa,

we sleep on the ground with children. It's very cold, we don't eat well… There are no clothes for children, nor for adults, and … there is no space to play… In the women's room, there were twelve beds, and there were 40 people in there.

[TbA]

In these conditions, it is unsurprising that women often develop ailments like dermatoses, scabies, and even chickenpox (as reported by Astrid, a detained woman we interviewed). The unsanitary environment was particularly concerning for pregnant women and children, who ideally should not be detained at all (UN High Commissioner for Refugees, 2012). Additionally, healthcare in detention—both at the UHSA and in Lisbon's “waiting zone”—was rather scarce. Medical assistance was provided by MdM staff voluntarily and only on certain days. When healthcare personnel were unavailable, detained people could only access basic medication (typically paracetamol) from center staff, or, in severe cases, be taken to the local hospital emergency room. However, according to our protagonists, staff often dismissed their complaints, fostering a sense of neglect and disregard for their wellbeing. As Delfina remarked,

You only have the right to go to the hospital if you are really feeling very unwell, if you are, let's say, really [feeling] terrible … when they see you are almost fainting or falling to the ground and dragging yourself along.

[TbA]

One of the most distressing accounts of neglect concerns Georgette, who suffered a miscarriage while detained in Lisbon's “waiting zone.” When this happened, she received no immediate assistance from center staff. She was left alone to cope with the traumatic event until her eventual release when she could finally consult a doctor. Although she had opportunities to report the abuse, Georgette was too afraid, feeling unsafe in the center and fearing for the wellbeing of her loved ones—her elderly mother, husband, and child—whom she believed were vulnerable to Portuguese immigration authorities. Thus, 4 days after the miscarriage, when she encountered a “nice” Europol officer (as she described her), Georgette voiced her fear and desire to leave detention but did not detail the violations she had endured:

I said “I can't say, nor do I want to say” because I was afraid. I only said, “I don't know these people! I'm just facing difficulty. I've been mistreated since I entered this country!”… I said, “I'm very afraid to stay here, with these people who are controlling the center.”

The officer eventually offered to take Georgette to the hospital, but she refused. She would never leave her loved ones behind, in detention, she said; she would rather die instead. As Georgette's story illuminates, an overall sense of unsafety and fear reigns in these carceral environments, which constitutes *uncaring* spaces by design.^10^ Immigration officers, who oversee these sites, exploit these feelings to govern day‐to‐day life and exert power over those confined. Many women, for instance, expressed their fear of being deported back to the countries they had fled in search of sanctuary and a new life. The constant threat of deportation, repeatedly voiced by immigration officers, left them profoundly distressed. As Ayda explained, “We don't know [why] all of [the] time, twice, they [immigration officers] said [to] us, ‘They want to deport you to Iran’. If … we go to Iran … it's dangerous for us.”

This vulnerability was exacerbated by a lack of information on several fronts, including the reasons for women's detention, transfers between centers (e.g., from Lisbon's “waiting zone” to the UHSA), daily life in detention, and, crucially, laws and procedures governing immigration and asylum in Portugal—often requiring Francesca to provide explanations. There was also a gap in information about women's basic rights, including the right to legal assistance. As Angela noted,

But they never said to me like that: “You don't have rights; you don't have rights.” They explained to me that you can even have a lawyer, but it's a waste of time … that I could have any lawyer if I was willing to pay.

[TbA]

Access to legal representation, or rather the lack of it, was a recurring justice‐related theme in the accounts of our protagonists. Most of the women we spoke with (14 of 25), along with many of those Francesca met inside the UHSA, reported having no legal assistance while detained, a situation which left them highly vulnerable to state and police abuse. Women seeking asylum in Portugal received support from the CPR for their appeals against rejection decisions, which were systematically applied to everyone. However, CPR acknowledged that this support was provided in a highly challenging context, characterized by significant constraints, such as shorter application deadlines (4 days to lodge appeals), communication barriers, and delays in accessing interpreters (Carreirinho & Portuguese Refugee Council, 2024). The situation for those detained inside the UHSA improved slightly when an SJR staff member established an ad hoc protocol with Portuguese Social Security, creating a fast‐track process for legal aid for detained individuals who met the income‐related criteria (Francesca's field observations).^11^

Uncertainty and confusion reigned in this context, as women were largely unaware of the status of their immigration and asylum cases and whether they would be released and allowed to remain in the country or rather deported. Most of our protagonists, particularly those seeking asylum, knew they had to complete 60 days in detention but were uncertain about what would happen next. Others were unsure of the length of their confinement, as the information they received and their observations of others' experiences were inconsistent. As Olga commented,

[The Ukrainian translator] said [we have to remain in detention for] 60 days, and it depends … there are people who stay in [detention for] 60 days, there are people who stay only 30 [days], [and] there are people who stay 40 [days].

[TbA]

This situation caused significant distress, particularly for women who did not speak Portuguese, as interpreters were rarely available—except for official interviews, such as asylum requests, and even then, not always. In daily life in detention, English was commonly used to communicate with non‐Portuguese speakers, including in written documents (e.g., the center's rules), even though many had a limited understanding of the language. When English was not an option, other detained women were often asked to assist with translation, as was the case with Khadija, who a fellow detainee (Adama) helped.

Such a mode of managing information in detention—where information is either not provided in a fully comprehensible manner (as in Khadija's case), fragmented (as in Makeda's case), or even contradictory (as in Angela's and Ayda's cases)—can be understood as a *politics of ignorance* designed to keep women uninformed and unaware (from the Latin *ignorāre*, derived from the Indo‐European *nōscere*, Cortellazzo & Zolli, 1999), including of existing rights and resources. This politics of ignorance consistently emerged in women's narratives, shaping their detention experiences throughout. Yet, as discussed in the next section, despite enduring the harshest conditions, women were neither passive victims nor mere spectators of the system's violence; instead, they strived to survive and resist.

### Survival, resistance, and protagonism: Creating bottom‐up ecologies of knowledge

Amidst the complexities of power and injustice that define life in these custodial institutions, women actively navigated, made sense of, and survived detention, as their testimonies reveal. In doing so, they also challenged the racialized, gendered, sexualized, and class‐based structures underpinning this oppressive system (Esposito et al., 2020).

Through their accounts, which illuminated the workings and intersectional violence of the detention regime, women offered powerful counternarratives to the official stories told from the perspective of the state and its proxies, reclaiming their humanity and dignity against a system designed to strip people of their personhood and meaning in life (Boochani, 2019). These counternarratives also crucially challenged the language embedded in the system's power structures. This is evident in Georgette's case, where she resisted the criminalizing rhetoric of Lisbon airport immigration officers, who accused her and her family of committing a crime by entering the country with fake passports:

They said, “You entered, used fake passports, and you committed a very serious crime, which leads to imprisonment!” And I was just shocked, “But, you know, we are Syrians, and you know about the situation in Syria…” I looked at him. “Excuse [me], Sir, but are we in 2014? And you don't know where we are on the map to understand what's going on in Syria!.”

[TbA]

Inside detention, women relied on various strategies to survive and keep their spirits up. For instance, some focused on the transitory nature of detention, adopting the mindset: “It will pass” (Ester) [TbA], framing the experience as a challenge to overcome. Others, like Makeda, coped by maintaining a positive outlook: “I am too young; I have to get married. I have to have children. I have to give them the best… This [detention] will not stop me from having those positive thoughts.” In some cases, women tried to reassure themselves that, despite the hardships, it was safer to be in detention than to return to their countries of origin. This sentiment particularly resonated with those who had come to Portugal seeking sanctuary, as in the case of Khadija. Some women we met, including Marcia, Ester, and Vanessa, spoke about trying to “normalize” this abnormal experience. For them, it was about being strong: “You have to endure, you just have to endure” (Ana) [TbA]. “Every day, I mark one,” emphasizing the temporal violence of detention. Most women hoped that this *stolen time* (Khosravi, 2018) could, at least in part, be “repaid” by the chance to build or continue their lives in Portugal.

Overall, reliance on faith emerged as a primary strategy for most women, regardless of their religious background. Faith helped them make sense of their experiences and maintain hope for a better future after detention. It served as a source of comfort amid such distressing circumstances, offering solace, guidance, and strength while bolstering the women's inner resources to persevere. In the absence of a dedicated faith room, detained women often prayed or read religious texts in their rooms. In some cases, they gathered for this purpose, cementing a sense of community and solidarity through their spiritual connection. These moments became instances of freedom and resistance against the daily oppression of detention life. As Makeda put it: “it's a mixture—Christians, Muslims, everyone, different groups—so we pray! If you find someone you can understand, pray together.”

In detention, as they waited for time to pass, women sought ways to distract themselves and avoid constantly thinking about their immigration cases. To do so, they engaged in various activities, such as watching TV in the common area, listening to music, reading, playing ping pong, chess, or board games, painting, exercising, playing football, walking, sunbathing, talking on the phone (during the 2 hours per day allowed for phone use), braiding hair (especially among African women), and chatting with others. Relationships among those detained were particularly meaningful, providing support on multiple levels. Many women emphasized how crucial the emotional support from other detained women was. They could talk, share stories, laugh, vent, and gain strength to face the harsh reality of detention. As Makeda summarized, “You talk, you cry, you share.”

Regardless of their diverse backgrounds and experiences, women often expressed a sense of “everyone being in the same boat” (Priscilla) [TbA]. This does not mean that divisions, tensions, and conflicts were absent; rather, it suggests that, at times, these were transcended in favor of a shared sense of belonging to a single “community of detainees” (see also Bosworth, 2014). In this context, diversity could become an asset rather than a barrier. For example, some women, like Ester, found that a silver lining in their difficult experience was meeting people from other countries they might not have encountered otherwise and forming new friendships, as Makeda noted. In some cases, like with Julia, it was even possible to find love in such a dehumanizing environment. Her encounter with a detained young man provided her with renewed energy and meaningful emotional support, which was crucial for her survival. As she highlighted, “He makes me feel very good because if it wasn't [for him]… I would already be depressed.” [TbA]

Relationships amongst those detained also served as a crucial source of informational support, as women often shared their knowledge of rights, procedures, and strategies to challenge the detention and deportation system. Such mutual support and solidarity were especially important in addressing the severe informational injustice discussed earlier (see Justice). Women with more time in detention or greater knowledge of the system would pass on their insights through word of mouth. This exchange of information covered not only the center's rules and how to navigate daily life in detention but also immigration and asylum proceedings, thereby creating an ecology of knowledge from “the bottom up.”

Forms of material support and solidarity were central to life inside the UHSA. Detained women would lend each other's mobile phones and share internet data, enabling everyone to contact their families and lawyers. They would also share clothes, cigarettes (if they smoked), and food (if they were receiving visits from the outside and the food was allowed to enter). Priscilla stated, “Why would I have a Coca‐Cola by myself … sometimes you feel like it, [and] I offer it to everyone” [TbA]. Women's solidarity was the primary form of resistance against this violent system. Moments of sharing and community building often took place at night when women gathered to talk before sleeping. In this context, the transphobic practice of isolating trans women in a separate area at night takes on particularly violent connotations. As Priscilla explained,

I don't want privileges—a room with a bathroom, nothing like that. I want to be like everyone else!… At night… I feel a bit separated [from the other women]… you're estranged from everyone… I feel alone.

At times, women also sought moments of isolation and meditation, especially when coping with difficult emotions, stress, and sadness. As Makeda commented, “It depends on the moods. Sometimes, you're blue, and you want to stay by yourself.” Some women intentionally avoided complaints and confrontations, fearing that any open interrogation of the system would jeopardize their cases. Priscilla, for example, told us she even refrained from asking for a blanket when cold, believing that staying quiet and avoiding “trouble” would improve her situation. Despite experiencing transphobic comments from some of the men detained with her, Priscilla chose not to report them, fearing the repercussions. “Sometimes I'm afraid that these things will count against me later” she explained. “If I cause trouble, it will mess up my life even more.” (Priscilla) [TbA]

Overall, the pervasive sense of arbitrariness and lack of accountability in the detention environment led some women to feel it was preferable to endure silently while awaiting their release. This attitude reflected not a lack of criticism toward the system, but rather a survival mechanism in such dire circumstances. Many women had to avoid deportation at all costs, fearing for their lives if forced back. Consequently, they utilized every available means for this purpose, including the limited legal tools provided by the system. To challenge their deportation, some of our protagonists sought international protection (as did Angela and Ana) or claimed their right to stay on health or family grounds (e.g., Julia and Ester). For women with Portuguese partners, marriage became a potential route, even if it was not an option they would have chosen under different circumstances. Others turned to their children—whether born or yet to come—to resist their expulsion.

Although Portugal legally recognizes same‐sex families, these cases—primarily involving cisgender women—can be seen as a strategic adaptation to heteropatriarchal norms that recognize women only as wives and/or mothers, or alternatively, as victims (on how border regimes reinforce heteronormativity, see Luibhéid & Chávez, 2020). Illustrative is the story of Julia, a 21‐year‐old Brazilian woman who was detained for 2 weeks while 4 months pregnant at the time of our encounter. Julia used her identity as a “pregnant woman” to highlight the system's unfairness and to expose detention centers as *uncaring* environments. She reported inadequate support for pregnancy‐related issues, such as morning sickness, and claimed her vulnerability was ignored, with staff refusing to take her to the hospital. Julia viewed the medical certificate for risky pregnancy as a key tool for opposing her deportation, particularly given the risk of the Zika virus in Brazil. As she said, “I can't suffer like this. It is no good for me; this is no good for the baby because I feel it and he feels it too.” (Julia) [TbA]

Similar to Julia, some women openly defied the system to contest injustice. As in Georgette's case, such resistance could manifest in verbal confrontations with staff members. The immigration officer managing the Lisbon “waiting zone” shouted at her without explaining what she had done wrong. Years later, after her release, Georgette encountered the same officer again, this time in her role as a cultural mediator. The officer expressed regret, claiming she was simply “doing her job.” Georgette responded courageously, saying, “Your job is to control the center, *not to break the law* [our emphasis]. I didn't know this was your job.” In other instances, women resisted by disobeying unjust rules and challenging violent practices, as Agatha did by refusing to take sedatives in detention (see Singer et al., 2022).

As Lisette described, some people also attempted to escape detention, with varying levels of success. In some instances—though less commonly than in other contexts (Esposito et al., 2019)—detention centers were damaged. For example, in August 2020, the Porto Airport “waiting zone” was “temporarily” closed following protests and acts of resistance by detained people, leading to significant damage (Mecanismo Nacional de Prevenção, 2021). Importantly, some women also chose to use their lived experiences to challenge the invisibility of this form of racialized state‐sanctioned violence, speak up to power and bring about change. As Georgette, who remained in Portugal and built a life there, meaningfully stated,

my son said, “Someone has to do something to close these centers!”… Only at that moment … did I decide to speak up… in the *Expresso* [Portuguese newspaper]. After that reportage I did, many people started talking to me … they told me that they didn't know what, what goes on in there [detention].

Beyond sharing her story publicly, Georgette decided to assist others seeking sanctuary in Portugal. Her detention experience, marked by violence, lack of support and language barriers, motivated her to become a catalyst for meaningful change on the ground, day by day.

## DISCUSSION

### Amplifying insights from women's lived experiences

The story emerging from our analysis highlights the violence inherent in Portugal's immigration detention regime, focusing on the complex struggles women face within these oppressive environments. Adopting a justice‐centered community psychology ecological framework requires an understanding of how these struggles are shaped by intersecting vectors of oppression, including “race,” gender, class, citizenship, and immigration status. However, our analysis moves beyond simply addressing the abusive nature of the detention system and its impacts on the (cis and trans) women we met. Instead, we aimed to uncover the linkages between women's lived experiences—articulated through their rich firsthand accounts—and the historical, sociopolitical, and cultural structures and processes that make detention and deportation possible in the first place (Dutta et al., 2024; Martín‐Baró, 1996). In doing so, we sought a nuanced understanding of the relationship between structural and institutional violence and injustice on one hand, and detained women's various forms of survival, resistance, and protagonism, on the other. Georgette's statement, “Who's breaking the law … not us, them!”—which we have chosen as the title of this paper—captures this complex relationship and underscores women's protagonism in challenging the abnormal reality of detention and revealing the intricacies of its violence.

The oppressive qualities of detention manifest in the relentless passage of time— which becomes emptied of any purposeful meaning, merely punctuating a stagnant existence (e.g., Griffiths, 2014; Khosravi, 2018). In this context, the reference points necessary for preserving a sense of self, envisioning a future, and maintaining vital connections to relationships and meanings are eroded. As the literature indicates (see, inter alia, Bosworth et al., 2018), detention not only dehumanizes those affected—women in our case—but, as our analysis reveals, also erodes their sense of personhood, aiming to reduce them to mere “bodies to be removed.” As Bosworth (2014) puts it, detention centres are *sites of estrangement*. Poor living and unsanitary conditions, coupled with a lack of medical and legal assistance and isolation from the outside world, are common features of the Portuguese detention centers we analyzed (for similar findings in other contexts, see Canning, 2017; Esposito et al., 2019; Lousley & Cope, 2017). Such a scenario—characterized by control and discipline alongside abandonment and neglect as mechanisms of power—created fertile ground for abuses to proliferate, hidden from public scrutiny. Uncertainty and fear pervaded the experiences of the women we met, who felt vulnerable in the face of state and immigration police powers (Bosworth, 2014; Griffiths, 2014). Overall, women's accounts reveal that detention centers are *uncaring* environments where they felt threatened and unsafe. In this context, their needs and experiences were systematically disregarded, jeopardizing their wellbeing. This systemic and institutional violence manifested in the mundane activities of daily life rather than in overt, high‐profile events—though such events did occur, as evidenced by Ihor Homeniuk's brutal death in 2020. Such violence was also intensified by the lack of information and advice—particularly legal advice—available to those in detention (see Bosworth & Kellezi, 2017).

In their psychogeographical countermapping study of migrant detention sites, Manek et al. (2023) define immigration detention as a “necropolitical space of [un]care” that obstructs the reproduction of a dignified life. Specifically, they identify four core mechanisms at play in these sites: [un]care, [un]safety, [dis]information, and [dys]functionality. These observations resonate with our findings. In our case, women described the system's response—embodied by those responsible for its operations—as characterized by “minimal” and “ordinary” actions: minimizing, mystifying, and avoiding direct naming. Many of our protagonists either did not speak, or only partially spoke Portuguese (the primary language used in daily life in detention). This situation and the lack of interpreters relegated them to a condition of structural “voicelessness” (see also Bosworth & Kellezi, 2014). Furthermore, even for those proficient in the language, the means for women to “make their voice heard” by the system were often denied, as evidenced by the lack of access to legal representation that we documented. When women did speak out, or when their bodies manifested their complex suffering (e.g., through illness or, as in Georgette's case, a miscarriage), a systematic pattern of denial and mystification ensued.

Overall, we argue that a politics of ignorance emerged from both the women's firsthand accounts and our observations of the detention system, leaving those trapped within these custodial sites in a state of profound uncertainty and confusion. This ignorance was fostered through the concealment, distortion, and fragmentation of key information and the provision of incomplete or inaccurate details about the system's rules and procedures. Combined with limited access to legal advice (see Bosworth & Kellezi, 2017), this strategy maintained women unaware of their rights and available resources, ultimately allowing state actors and their proxies to govern more effectively while stifling potential resistance.

Although this situation made it challenging for women to navigate the system and daily life within it, they still found multiple creative ways to survive and resist. Buckingham et al. (2021, p. 271) define resistance as “engagement in any action that undermines oppressive power structures, regardless of intent or outcome.” According to them, resistance can take various forms—active or passive, organized or unorganized, overt or covert. It can range from social movements to everyday acts of defiance, and it can be individual or collective, with diverse targets and purposes. In the context of the detention center where our research took place, the daily resistance of detained women was expressed through various strategies and praxes. In particular, in response to the everyday dehumanization and violence they faced, women created “counterspaces” (Buckingham et al., 2021; Case & Hunter, 2012) to foster a sense of community and restore their sense of self, meaning, and purpose. In doing so, they forged the strength needed to withstand and resist the detention and deportation systems from below. The solidarity and mutual support within these “counter spaces”—often formed in women's dormitories or during brief moments of respite from the system's control—were vital for surviving this oppressive environment, facilitating the exchange of emotional, spiritual, material, and informational support. Information pooling was especially crucial for challenging the politics of ignorance underpinning the Portuguese detention and deportation system. This information encompassed the center's rules, daily life, immigration and asylum proceedings, and strategies for accessing rights and resisting injustice, ultimately fostering an ecology of knowledge and resistance from the bottom up.

As with any research, there are limitations to this work and opportunities for further enrichment. Detention centers are oppressive and distressing environments where individuals face uncertainty about their futures and largely feel uncared for and unsafe. These conditions inevitably impact any any action‐research process processes in such settings, limiting the potential for more participatory modes of engagement (Bosworth, 2014; Bosworth & Kellezi, 2017). Building a foundation of “just enough trust” is essential for enabling detained people to freely express their views and experiences without fear of repercussions; this process requires considerable effort and a radical commitment to meaningful relational engagement. As Bosworth and Kellezi (2017, p. 126) note in reflecting on their study within British detention centers, “asking for too much detail could make women and men feel as though they were undergoing another Home Office interview.” This was also true in the context of our research. That is why it was crucial for women to understand that we were firmly on their side. The “hyperdiversity” that typically characterizes detention environments further complicated the scenario (Bosworth, 2014). As mentioned earlier, we are all cisgender, White, Western, educated women with no direct experience of immigration detention (though some of us have experienced immigration controls). Our perspectives are shaped by our positionalities, despite our commitment to critically acknowledging and repositioning our privilege to build intersectional solidarities with those on the frontlines of border struggles.

Finally, this research does not aim for generalizability; instead, we embrace the situatedness of our work. Nonetheless, we believe certain insights may resonate across contexts, given the similar ways in which immigration detention systems operate across jurisdictions. Future studies could further enrich our findings by documenting dynamics of power, violence, survival, and resistance in other detention settings, adapting and expanding the justice‐centered ecological framework used here. Including perspectives from detained men, queer and gender‐nonconforming individuals, as well as practitioners and activists, could deepen our understanding of these systems and their oppressive qualities. Additionally, creative participatory approaches and methodologies could provide these protagonists with more effective means to articulate their complex experiences, facilitating the countermapping of the insidious forms of violence at play within detention centers and the various strategies of resistance against them. Ultimately, involving people with lived experience of detention as co‐researchers could significantly enhance our understanding of immigration detention and promote social justice throughout the research process.

## CONCLUSIVE REMARKS: ALL THAT REMAINS TO DO

Violent b/ordering logics and practices profoundly shape our everyday lives and will continue to do so in the foreseeable future. Immigration detention and deportation stand as extreme manifestations of such violent b/orders, ultimately reinforcing an exclusionary politics of belonging that dictates the unequal distribution of life chances among different population groups (Atallah & Dutta, 2022; Esposito et al., 2024). Building on recent calls from community‐engaged scholars and activists to challenge border and citizenship regimes, along with their colonial underpinnings (Dutta et al., 2024), we conclude with some insights into how community psychologists, inspired by critical, decolonial, and liberation perspectives, can forge solidarities‐in‐action with communities at the forefront of struggles for the freedom to move, stay, and live free from border violence. These insights, informed by our findings, transcend individual or context‐specific concerns. Importantly, the struggles of our protagonists must be understood in relation to the structural, symbolic, and material forces that sustain the racialized and unjust social orders in which we live. In essence, the suffering of detained women has political roots (Bosworth, 2023). Hence, our response to this suffering—and how we engage with it—is inherently political.

The veil of opacity enveloping detention centers—where violence is perpetuated in mundane ways—is intentional. It seeks to conceal what happens within these often remote and inaccessible sites from public scrutiny. The politics of ignorance that saturates daily life in detention, shaping women's experiences vis‐à‐vis state and institutional powers, serves a complementary function. It disrupts meaningful connections between the inside and outside, hindering the flow of information and solidarity that could empower those detained to resist this unjust system. Overall, it obstructs accountability for state‐sanctioned racialized violence and curtails the formation of effective intersectional solidarities to challenge it.

In such oppressive environments, research can play an important role, even if it involves bearing the risk of losing academic privileges and future institutional access. As Kirmayer et al. (2004, p. 87) put it, research can constitute “a form of civil disobedience.” By entering these highly politicized sites as activist researchers and psychosocial accompaniers, we can leverage our privilege to serve those detained, supporting their bottom‐up ecologies of knowledge and resistance. Guided by an ethic of solidarity and equipped with information and resources to share, we can amplify detained people's efforts for survival and system contestation. We can also bear witness to their struggles and report abuse and violations to the outside. Overall, our efforts should be twofold: first, countering the politics of ignorance that hypervulnerabilizes those detained, and second, challenging the politics of organized forgetting (Esposito & Caja, 2025) that obscure the racialized (classed and gendered) state‐sanctioned violence at the borders and its colonial foundations.

Through our writing, we can contribute to challenging the erasure of these sites of persistent injustice (Prilleltensky, 2012) from public consciousness. More importantly, we can support demands for justice, accountability, and, ultimately, detention abolition. By visiting people in detention, as the visitors' movement does (see, for instance, https://www.aviddetention.org.uk), and building meaningful relationships of solidarity, we can challenge the state's violent negation of their social personhood, which seeks to render those confined within these sites “nameless” and “voiceless.” Community psychologists and other community‐engaged scholars must begin to address this issue, using their skills, knowledge, and resources to challenge these “pervasive pathologies of power” (Kirmayer et al., 2004, p. 85). This contribution serves as an invitation to start doing so. As Georgette urges us to recognize, it is “them”—the state and supranational powers, along with their institutional and legal apparatuses—who are truly “breaking the law.” We must be resolute in defending this truth. This responsibility is rooted in moral, ethical, and political considerations, as well as in a shared sense of humanity. Simply put, we must strive to render borders and detention centers obsolete (Davis, 2003), working toward futures defined by freedom, dignity, and justice for all.

## CONFLICT OF INTEREST STATEMENT

The authors declare no conflicts of interest.

## Footnotes

1 We understand the term “women” as referring to a plurality of subjectivities defined by intersecting axes of power and difference, such as race, class, sexuality, ability, citizenship, and immigration status. According to Sara Ahmed (2017, p. 14), this umbrella category, “those who travel under the sign *women*,” encompasses both those recognized as women by society and those not recognized as women despite their self‐identification, including transgender, cisgender, endosex, and intersex women.

2 The term “politics of organised forgetting” has been used by some of us to characterize the manufactured opacity surrounding the Portuguese detention system. This politics is evident in the erasures, absences, and silences that define dominant narratives in the field. Such opacity, combined with the erasure of the complex connections between the country's colonial history and the contemporary “making of migration,” is central to the harmful structure of the national border regime and serves to perpetuate the racial hierarchies and violence upon which the national social order is historically built (for further reading, see Esposito & Caja, 2025).

3 An *Espaço Equiparado a Centro de Instalação Temporária* (EECIT) is defined by Law 23/2007 of July 4 as “a designated space created in the international zone of a Portuguese airport for the *installation* [emphasis added] of passengers not admitted into the national territory who are awaiting reboarding.” According to the Asylum Information Database report (Carreirinho & Portuguese Refugee Council, 2024), the current capacities of Portuguese EECITs are as follows: Lisbon, 24 places; Porto, 18 places; Faro, 13 places; Funchal and Ponta Delgada, data unavailable.

4 It is unknown how many of these individuals are actually transgender or gender‐nonconforming people, as detained people are forced into binary and heteronormative categories for classification purposes.

5 Until October 29, 2023, date in which it was extinguished, in Portugal, the police force tasked with immigration enforcement powers was the Immigration and Border Service (*Serviço de Estrangeiros e Fronteiras‐SEF*). After that date, SEF functions were redistributed to other police and armed forces, including the Public Security Police (*Polícia de Segurança Pública*) and the National Republican Guard (*Guarda Nacional Republicana*). These two are currently in charge of managing the existing CIT and EECITs, depending on the jurisdiction in which these centers are located.

6 We align with a common stance in critical migration studies that advocates for the use of the terms “illegalized” and “illegalization” when referring to undocumented individuals. While no person can be deemed “illegal,” these terms underscore the sociopolitical construction of migrants’ illegality through laws and policies, as well as the influence of various social actors, contexts, and practices in this process (De Genova, 2002).

7 This perspective points to the constitutive role of nation‐states’ bordering practices in the enactment of some people as “migrants”, and the role that “race” and color lines play in this process (see, e.g., Tazzioli, 2020).

8 Ethical approval for this study was granted by the ISPA‐Instituto Universitário Research Ethics Commission and the Social Sciences and Humanities Interdivisional Research Ethics Committee at the University of Oxford (Ref No.: R57016/RE001).

9 F. D. C. and F. E. are, respectively, a permanent and a precarious researcher, while E. B. is a doctoral candidate, and F. D. L. is a postgraduate specialization student.

10 In line with critical scholarship (e.g., Manek et al., 2023; The Care Collective, 2020), we use here the term “uncaring” to describe a condition in which individuals or communities are systematically denied care. In this case, an “uncaring environment” refers to a setting characterized by institutional neglect and/or systemic indifference.

11 State‐funded legal aid in Portugal is available to everyone, both inside and outside detention, ensuring access to legal representation for those unable to afford a private lawyer. Managed by the Portuguese Social Security, eligibility for state‐funded legal aid is determined by the *Indexante dos Apoios Sociais* (Index of Social Support), with an income threshold of €509.26. The protocol established by SJR staff enabled detained individuals meeting this criterion to be assigned a state lawyer in a relatively short time—as this process typically takes much longer, often exceeding the statutory upper time limit for detention of 60 days.

## APPENDIX 1

### 1.1

**Table A1:** The protagonists in our research.

	Range	Mean
Age (years)	18–70	34.1
Time spent in Portugal (days)	10–6205	474
Detained on arrival	15	–
Time spent in detention (days)	4–57	29.64
	*N*	%
Gender self‐identification		
Cis woman	23	92
Trans woman	2	8
Region of citizenship		
Central Africa	9	36
Latin America	6	24
West Africa	4	16
Eastern Europe	3	12
Middle East	2	8
Asia	1	4

## APPENDIX 2

### 2.1

**Table A2:** The analytical framework based on Kelly (2006) and Prilleltensky's (2012) principles.

Interdependence	Examining how the life space of detained people and practitioners working at these sites, as well as their person‐environment interdependences, are disrupted by immigration detention oppressive qualities
Cycling of resources	How resources (e.g., money and goods but also services and expertise) are created, managed, and allocated in the detention system, and the role played by policies and politics, along with other forces, in this process
Adaptation	Analysis of the strategies that both detained people and practitioners working at these sites employ to survive and resist their daily life in detention
Succession	Temporal understanding of the detention system and social life within it—referring to time and temporalities in detention as bound up in power relations
Justice	Referring to the oppressive and dehumanizing mechanisms at play in the detention environment

## References

[ajcp12784-bib-0001] Ahmed, S. (2017). Living a feminist life. Duke University Press.

[ajcp12784-bib-0002] Atallah, D. G. , & Dutta, U. (2022). “Creatively in coalition” from Palestine to India: Weaving stories of refusal and community as decolonial praxis. Journal of Social Issues, 78(2), 434–451. 10.1111/josi.12460

[ajcp12784-bib-0003] Bachmann, C. L. (2016). No safe refuge: Experiences of LGBT asylum seekers in detention. UK Lesbian & Gay Immigration Group. https://tinyurl.com/yc4p848b

[ajcp12784-bib-0004] Bashford, A. , & Strange, C. (2002). Asylum‐seekers and national histories of detention. Australian Journal of Politics & History, 48(4), 509–527. 10.1111/1467-8497.00273 15889508

[ajcp12784-bib-0005] Boochani, B. (2019, July 30). *Writing is an act of resistance* [Video]. YouTube. https://l1nk.dev/upiNn

[ajcp12784-bib-0006] Bosworth, M. (2014). Inside immigration detention. Oxford University Press.

[ajcp12784-bib-0007] Bosworth, M. (2023). The politics of pain in immigration detention. Punishment & Society, 25(2), 307–323. 10.1177/14624745211048811

[ajcp12784-bib-0008] Bosworth, M. , Fili, A. , & Pickering, S. (2018). Women and border policing at the edges of Europe. Journal of Ethnic and Migration Studies, 44(13), 2182–2196. 10.1080/1369183X.2017.1408459

[ajcp12784-bib-0009] Bosworth, M. , & Kellezi, B. (2014). Citizenship and belonging in a women's immigration detention centre. In C. Philips & C. Webster (Eds.), New directions in race, ethnicity and crime (pp. 80–96). Routledge.

[ajcp12784-bib-0010] Bosworth, M. , & Kellezi, B. (2017). Doing research in immigration removal centres: Ethics, emotions and impact. Criminology & Criminal Justice, 17(2), 121–137. 10.1177/1748895816646151

[ajcp12784-bib-0011] Braun, V. , & Clarke, V. (2006). Using thematic analysis in psychology. Qualitative Research in Psychology, 3(2), 77–101. 10.1191/1478088706qp063oa

[ajcp12784-bib-0012] Braun, V. , & Clarke, V. (2019). Reflecting on reflexive thematic analysis. Qualitative Research in Sport, Exercise and Health, 11(4), 589–597. 10.1080/2159676X.2019.1628806

[ajcp12784-bib-0013] Buckingham, S. L. , Langhout, R. D. , Rusch, D. , Mehta, T. , Rubén Chávez, N. , Ferreira van Leer, K. , Oberoi, A. , Indart, M. , Paloma, V. , King, V. E. , & Olson, B. (2021). The roles of settings in supporting immigrants' resistance to injustice and oppression. American Journal of Community Psychology, 68(3–4), 269–291. 10.1002/ajcp.12515 33960422 PMC9290340

[ajcp12784-bib-0014] Canning, V. (2017). Gendered harm and structural violence in the British Asylum System. Routledge.

[ajcp12784-bib-0015] Carreirinho, I. , & Portuguese Refugee Council . (2024). Portugal Country Report. 2023 update. Asylum Information Database. https://asylumineurope.org/reports/country/portugal/

[ajcp12784-bib-0016] Case, A. D. , & Hunter, C. D. (2012). Counterspaces: A unit of analysis for understanding the role of settings in marginalized individuals' adaptive responses to oppression. American Journal of Community Psychology, 50(1–2), 257–270. 10.1007/s10464-012-9497-7 22374370

[ajcp12784-bib-0017] Chicco, J. , Esparza, P. , Lykes, M. B. , Balcazar, F. E. , & Ferreira, K. (2016). Policy statement on the incarceration of undocumented migrant families. American Journal of Community Psychology, 57(1–2), 255–263. 10.1002/ajcp.12017 27217327

[ajcp12784-bib-0018] Clarke, V. , & Braun, V. (2017). Thematic analysis. The Journal of Positive Psychology, 12(3), 297–298. 10.1080/17439760.2016.1262613

[ajcp12784-bib-0019] Cortellazzo, M. , & Zolli, P. (1999). DELI—Dizionario Etimologico della Lingua Italiana. Zanichelli.

[ajcp12784-bib-0020] Davis, A. (2003). Are Prisons Obsolete? Seven Stories.

[ajcp12784-bib-0021] Demony, C. (2021, May 21). Portugal border cops sentenced to jail over death of detained traveller. *Reuters*. https://encr.pw/5Fpw4

[ajcp12784-bib-0022] Dutta, U. , Azad, A. K. , & Tanjeem, N. (2024). Examining citizenship regimes in Assam through a structural and cultural violence lens. American Journal of Community Psychology, 73(1–2), 294–311. 10.1002/ajcp.12715 37925615

[ajcp12784-bib-0023] Dutta, U. , Sonn, C. C. , & Lykes, M. B. (2016). Situating and contesting structural violence in community‐based research and action. Community Psychology in Global Perspective, 2(2), 1–20. 10.1285/i24212113v2i2p1

[ajcp12784-bib-0024] Edge, I. , Kagan, C. , & Stewart, A. (2003). Living poverty in the UK: Community psychology as accompaniment. https://tinyurl.com/mr8zxwf2

[ajcp12784-bib-0025] Esposito, F. , & Caja, E. (2025). Detention and deportation in Portugal: The colonial legacies of a racial governing of mobility. In G. Fabini , C. F. Bessa , V. Ferraris , & J. Brandariz (Eds.), Border criminologies from the periphery: Cross‐national conversations on border penality. Routledge.

[ajcp12784-bib-0026] Esposito, F. , Matos, R. , & Bosworth, M. (2020). Gender, vulnerability and everyday resistance in immigration detention: Women's experiences of confinement in a Portuguese detention facility. International Journal for Crime, Justice and Social Democracy, 9(3), 5–20.

[ajcp12784-bib-0027] Esposito, F. , Ornelas, J. , Briozzo, E. , & Arcidiacono, C. (2019). Ecology of sites of confinement: Everyday life in a detention center for illegalized non‐citizens. American Journal of Community Psychology, 63(1–2), 190–207. 10.1002/ajcp.12313 30758839

[ajcp12784-bib-0028] Esposito, F. , Rebelo, D. , Olanrewaju, M. , Vine, M. , Fernandes‐Jesus, M. , Bodden, D. , Kalokoh, A. , & Olson, B. (2024). A community psychology for migrant justice: Critically examining border violence and resistance during the COVID‐19 syndemic. American Journal of Community Psychology, 73(1–2), 27–43. 10.1002/ajcp.12669 37126214

[ajcp12784-bib-0029] Farmer, P. (2013). To repair the world: Paul farmer speaks to the next generation. University of California Press.

[ajcp12784-bib-0030] Flynn, M. (2012). Who must be detained? Proportionality as a tool for critiquing immigration detention policy. Refugee Survey Quarterly, 31(3), 40–68. 10.1093/rsq/hds008

[ajcp12784-bib-0031] Garraio, J. , Solovova, O. , & Santos, S. J. (2022). The (in)visibility of racialized border violence? A Ukrainian killed in Lisbon airport. State Crime Journal, 11(1), 90–109. 10.13169/statecrime.11.1.0090

[ajcp12784-bib-0032] De Genova, N. P. (2002). Migrant ‘illegality’ and deportability in everyday life. Annual Review of Anthropology, 31(1), 419–447. http://www.jstor.org/stable/4132887

[ajcp12784-bib-0033] Goffman, E. (1961). Asylums: Essays on the social situation of mental patients and other inmates. Anchor Doubleday.

[ajcp12784-bib-0034] Gorjão Henriques, J. (2018, September 3). Estrangeiras detidas no aeroporto de Lisboa estão ‘mais expostas’ a assédio, *Público*. https://l1nq.com/I16C6

[ajcp12784-bib-0035] Gorjão Henriques, J. (2019, April 10). SEF deteve três crianças no aeroporto, ministro pede investigação, *Público*. https://acesse.dev/TwYPp

[ajcp12784-bib-0036] Griffiths, M. B. E. (2014). Out of time: The temporal uncertainties of refused asylum seekers and immigration detainees. Journal of Ethnic and Migration Studies, 40(12), 1991–2009. 10.1080/1369183X.2014.907737

[ajcp12784-bib-0037] Human Right Watch . (2016). Do you see how much I'm suffering here?’ Abuse against transgender women in US immigration detention. https://tinyurl.com/bdcp22td

[ajcp12784-bib-0065] IOM Portugal . (n.d). *Mainstream human rights in detention center*. https://portugal.iom.int/mainstreaming-human-rights-detention-centers

[ajcp12784-bib-0038] Jurcik, T. , Sunohara, M. , Yakobov, E. , Solopieiva‐Jurcikova, I. , Ahmed, R. , & Ryder, A. G. (2019). Acculturation and adjustment of migrants reporting trauma: The contextual effects of perceived ethnic density. Journal of Community Psychology, 47(6), 1313–1328. 10.1002/jcop.22183 30981217

[ajcp12784-bib-0039] Kelly, J. G. (2006). Becoming ecological: An expedition into community psychology. Oxford University Press.

[ajcp12784-bib-0040] Khosravi, S. (2018). Stolen time. Radical Philosophy, 2(3), 38–41.

[ajcp12784-bib-0041] Kirmayer, L. J. , Rousseau, C. , & Crépeau, F. (2004). Research ethics and the plight of refugees in detention. Monash Bioethics Review, 23(4), S85–S92. 10.1007/BF03351423 15688516

[ajcp12784-bib-0042] Langhout, R. , Buckingham, S. L. , Oberoi, A. , Chavez, N. , Rusch, D. , Esposito, F. , & Suarez‐Balcazar, Y. (2018). Statement on the effects of deportation and forced separation on immigrants, their families, and communities. American Journal of Community Psychology, 62(81–2), 3–12. 10.1002/ajcp.12256 30066405

[ajcp12784-bib-0043] Lindberg, A. (2022). Deportation limbo: State violence and contestations in the Nordics. Manchester University Press.

[ajcp12784-bib-0044] Lousley, G. , & Cope, S. (2017). We are still here: The continued detention of women seeking asylum in Yarl's wood. Women for Refugee Women.

[ajcp12784-bib-0045] Luibhéid, E. , & Chávez, K. R. (2020). Queer and trans migrations: Dynamics of illegalization, detention, and deportation. University of Illinois Press.

[ajcp12784-bib-0046] Lykes, M. B. , Hershberg, R. M. , & Brabeck, K. M. (2011). Methodological challenges in participatory action research with undocumented central American migrants. Journal for Social Action in Counseling & Psychology, 3(2), 22–35. 10.33043/JSACP.3.2.22-35

[ajcp12784-bib-0047] Manek, J. , Nethery, A. , Esposito, F. , Pérez‐Sales, P. , & Horz, H. (2023). Mapping migration detention: Mixed methods, grounded theory, transdisciplinary encounters. Methods in Psychology, 9, 100129. 10.1016/j.metip.2023.100129

[ajcp12784-bib-0048] Marmot, M. (2004). Status syndrome: How your social standing affects your health and longevity. Bloomsbury.

[ajcp12784-bib-0049] Martín‐Baró, I. (Ed.). (1996). Writings for a liberation psychology. Harvard University Press.

[ajcp12784-bib-0050] McGregor, J. (2011). Contestations and consequences of deportability: Hunger strikes and the political agency of non‐citizens. Citizenship Studies, 15(5), 597–611. 10.1080/13621025.2011.583791

[ajcp12784-bib-0051] Mecanismo Nacional de Prevenção (MNP) . (2021). Relatório à Assembleia da República 2020. Provedor de Justiça. https://tinyurl.com/25e2tj9r

[ajcp12784-bib-0052] Prilleltensky, I. (2012). Wellness as fairness. American Journal of Community Psychology, 49(1–2), 1–21. 21643926 10.1007/s10464-011-9448-8

[ajcp12784-bib-0064] Riva, S. , Campbell, S. , Whitener, B. , & Medien, K. (2024). Border abolition now. Pluto Press.

[ajcp12784-bib-0053] Scheel, S. , & Tazzioli, M. (2022). Who is a migrant? Abandoning the nation‐state point of view in the study of migration. *Migration Policy Group*, *1*(002).

[ajcp12784-bib-0054] Singer, E. K. , Molyneux, K. , Kaur, K. , Kona, N. , Malave, G. S. , & Baranowski, K. A. (2022). The impact of immigration detention on the health of asylum seekers during the COVID‐19 pandemic. SSM—Qualitative Research in Health, 2, 100072. 10.1016/j.ssmqr.2022.100072 35340588 PMC8940268

[ajcp12784-bib-0055] Solano, G. , & Huddleston, T. (2020). Migrant integration policy index. Migration Policy Group. https://www.mipex.eu/

[ajcp12784-bib-0056] Tazzioli, M. (2020). The making of migration: The biopolitics of mobility at Europe's borders. Sage.

[ajcp12784-bib-0057] The Care Collective . (2020). The care manifesto: The politics of interdependence. Verso Books.

[ajcp12784-bib-0058] UN High Commissioner for Refugees (UNHCR) . (2012). Guidelines on the applicable criteria and standards relating to the detention of asylum‐seekers and alternatives to detention. Refworld. https://www.refworld.org/policy/legalguidance/unhcr/2012/en/87776

[ajcp12784-bib-0059] Watkins, M. (2015). Psychosocial accompaniment. Journal of Social and Political Psychology, 3(1), 324–341. 10.5964/jspp.v3i1.103

[ajcp12784-bib-0060] Von Werthern, M. , Robjant, K. , Chui, Z. , Schon, R. , Ottisova, L. , Mason, C. , & Katona, C. (2018). The impact of immigration detention on mental health: A systematic review. BMC Psychiatry, 18, 382. 10.1186/s12888-018-1945-y 30522460 PMC6282296

[ajcp12784-bib-0061] Wilsher, D. (2011). Immigration detention: Law, history, politics. Cambridge University Press.

[ajcp12784-bib-0062] Zou, J. , & Deng, X. (2021). The effect and mechanism of neighbourhood choice on socioeconomic integration of migrants: Evidence from China. Journal of Community Psychology, 49(2), 620–652. 10.1002/jcop.22483 33270919

